# Goal management training for adults with ADHD – clients’ experiences with a group-based intervention

**DOI:** 10.1186/s12888-021-03114-4

**Published:** 2021-02-19

**Authors:** Emilie S. Nordby, Sigrid Gilje, Daniel A. Jensen, Lin Sørensen, Signe H. Stige

**Affiliations:** 1 Department of Biological and Medical Psychology, Faculty of Psychology, University of Bergen, Bergen, Norway; 2 Division of Psychiatry, Haukeland University Hospital, Bergen, Norway; 3 Department of Mental Health and Addiction, Vestre Viken Hospital Trust, Kongsberg, Norway; 4 Betanien Hospital, Bergen, Norway; 5 Department of Clinical Psychology, Faculty of Psychology, University of Bergen, Bergen, Norway

**Keywords:** ADHD, Adults, Goal management training, Group-based interventions, Qualitative research

## Abstract

**Background:**

There is growing evidence for the efficacy of group-based interventions for adults with ADHD. However, there is still a lack of research investigating how clients experience participating in such interventions. The aim of the current study was to explore how adults with ADHD experience participating in a group-based intervention (Goal Management Training) for ADHD.

**Method:**

We conducted individual, semi-structured, interviews with ten adults with ADHD who had participated in Goal Management Training administered as a group intervention. The interviews were transcribed verbatim and analyzed using thematic analysis within a hermeneutic phenomenological framework.

**Results:**

Our analysis identified three main themes. *The participants’ starting point* captured the participants’ motivation and expectations prior to treatment. *The ambiguity of the group – the various meanings of the group* consisted of three sub-themes (*The group created a sense of belonging - “I am not alone”*; *The personal cost of participating in the group - “At times it was a hot mess”*; and *The group supported the learning experience - “We worked with it together”)*. *The group promoted positive change – How the group affected the participants’ everyday lives* consisted of two sub-themes (*Managing ADHD in daily life - “It’s much easier to handle everyday life”*, and *Personal growth - “Gaining new perspectives”*).

**Conclusion:**

The group format was experienced as a valuable aspect of treatment. The structure provided by Goal Management Training allowed participants to expand their perspectives and experience improved management of ADHD, as well as personal growth. The opportunity to exchange experiences with others in similar situations was seen as particularly beneficial and brought feelings of recognition and belonging. However, some also experienced the group as a burden at times, for instance by stealing one’s focus. This study expands existing knowledge by exploring clients’ experiences of participating in group-based interventions for ADHD and shows how the group format provided participants with more than they had hoped for. While expecting a more instrumental outcome of treatment, such as tools to manage ADHD, participants also gained a welcomed, but unexpected outcome of personal growth.

**Supplementary Information:**

The online version contains supplementary material available at 10.1186/s12888-021-03114-4.

## Background

Attention Deficit Hyperactivity Disorder (ADHD) is a persistent neurodevelopmental disorder marked by inattention, impulsivity and hyperactivity [1]. ADHD has an estimated prevalence of about 5% in children, and more than 50% of the children diagnosed with ADHD continue to show symptoms as adults [2, 3]. Findings from qualitative research indicate that adults with ADHD experience a wide range of consequences related to their diagnosis, such as distractibility, inattentiveness, impulsivity, restlessness, procrastination, and lack of motivation, which further impacts their day-to-day functioning in terms of execution of daily chores, educational goals, finances and occupational performance [4–6]. Moreover, ADHD is seen to affect the individual’s self-esteem and self-efficacy [7]. This is perceived as an additional burden, as it may contribute to withdrawal from society, social isolation and impaired psychosocial functioning [7]. Individuals with ADHD are also at higher risk of developing other psychiatric disorders, especially depression and anxiety [8]. Subsequently, ADHD is seen to affect a wide range of life domains, underscoring the importance of treatment approaches adopting a broad focus.

The most commonly offered treatment for adults with ADHD is medications [9]. The effectiveness of medications on ADHD symptomatology is well established [10, 11], but medications can also cause adverse effects, such as sleep problems, anxiety, dizziness, headaches and lack of appetite [10, 12]. In addition, some individuals do not respond sufficiently to medications, and many still have residual impairments [13, 14]. As such, both clinicians and the patient group advocates for a multimodal treatment approach, including both medications and psychological interventions [9, 15].

Research on treatment of ADHD has traditionally focused on pharmacological treatment, and less research has investigated psychological interventions, especially among adults. Current evidence on non-pharmacological treatment for adults with ADHD indicates that these interventions can lead to an improvement in self-reported symptoms of ADHD, where Cognitive Behavioral Therapy has the most evidence to current date [16, 17]. In particular, there is increasing evidence for the efficacy of group-based interventions for adults with ADHD [18–20]. Studies investigating group-based interventions for adults with ADHD have found an improvement in self-reported ADHD symptoms [18, 21], self-esteem [22], and self-efficacy [20] following treatment. The participants in these group-based interventions have also indicated an appreciation for the possibility of sharing experiences with their peers [18, 20].

Goal management training (GMT) is a group-based cognitive-rehabilitation approach that has been investigated as a potential non-pharmacological treatment for adults with ADHD with promising results [23]. The aim of GMT is to improve executive functioning, focusing especially on inhibitory control, through teaching problem-solving skills, inhibition strategies and mindfulness techniques for sustained and focused attention [24]. GMT is a structured approach, with weekly group sessions consisting of three main components; therapist tutoring, in-class exercises and discussions [24]. GMT is assumed to be a well-suited approach for ADHD as it concretely targets executive functioning, which is by many assumed to be the core deficit in ADHD [25]. The research on GMT as an intervention for ADHD is still limited, with one pilot study showing an improvement in cognitive functioning as rated by a clinician through a structured interview [23]. However, GMT has been shown to improve inhibitory control and executive functioning in other diagnostic groups, such as substance abuse [26], acquired brain injury [24], and spina bifida [27]. GMT has also been shown to improve other measures, such as emotional health and coping [28].

While some studies have reported that receiving treatment together in a group is an important and helpful aspect in psychological interventions for adults with ADHD [18, 20], no study has, to our knowledge, used qualitative methods to explore adult clients’ experiences with GMT or other group-based interventions for ADHD. An understanding of the individual’s experiences of the group and its impact might be a valuable research contribution in order to improve GMT, as well as other group-based approaches for ADHD. Moreover, a systematic study of clients’ experiences with GMT, using qualitative methods, will expand our knowledge base, as there are certain aspects of the clients’ experiences that will not be satisfactorily answered using only quantitative measures [29].

## Method

### Aim

The aim of the current study was to explore how adults with ADHD experience participating in a group-based intervention (GMT) for ADHD.

### Study setting

The data analyzed in this paper originate from an ongoing effect study that investigates GMT for adults with ADHD, led by the fourth author. There will be a separate paper reporting on the participants’ experience with the content and components of GMT, and other papers using quantitative data to investigate the effectiveness of the intervention. The third and fourth author will be responsible for these upcoming papers.

The GMT intervention consisted of nine weekly group sessions lasting 2 h each. The group sessions were centered around a different topic each week, such as inattentive errors, the autopilot, goal setting, decision-making, and planning [30]. Between the group sessions participants were given homework assignments that involved practicing techniques they had learned in class and logging situations where they were inattentive, forgetful, or made some sort of mistake in their daily routine.

### Study design and theoretical framework

The current study is a qualitative interview study of adults with ADHD who have participated in GMT. In our investigation we sought to capture the experiences of the participants as closely as possible, but also to be able to abstract common themes in the material. We therefore chose a qualitative approach, more specifically the ontological and epistemological framework of hermeneutic phenomenology [31, 32].

### Recruitment and participants

Participants in the GMT effect study were recruited through two approaches. First, a subset of participants from a larger multidisciplinary research project, entitled “ADHD: from clinical characterization to molecular mechanisms” (for description see [33]) were invited to participate by mail. Second, participants were recruited through distributing information material and giving short presentations at local outpatient clinics. Eligible participants had to have a confirmed ADHD diagnosis and be at least 18 years of age. Individuals that suffered from severe mental illness, such as lifetime psychosis, ongoing substance abuse or ongoing suicidality, were excluded from the study. Out of 32 included participants, 21 participants completed the treatment. All 21 participants who completed the treatment were approached at their last group session and invited to take part in a qualitative interview. Of the 21 invited, 10 accepted the invitation. Those who declined reported doing so due to busy schedules, not finding the time, thinking they could not contribute with much, or simply not having an interest. All participants were compensated with 1000 NOK to cover travel expenses upon completion of the follow-up assessment, regardless of whether they took part in the qualitative interview or not.

The participants that took part in the qualitative interviews included seven men, two women and one participant who did not identify as male or female. The participants’ age ranged from 21 to 49 years. Most were full-time employees or full-time students, and a few were part-time employees or part-time students. Almost all participants had been diagnosed with ADHD in adult age, and about half of them were currently using medications prescribed due to their ADHD. Several participants had received other psychological treatments during the past year, mainly related to ADHD. Some participants had comorbid disorders, with the most common being depression and anxiety disorders.

### Data collection

A total of 10 interviews were conducted. The interviews took place between November 2017 and April 2018 (all within 1 month after each participant’s completion of the treatment). The interviews were conducted at the Neuropsychological Outpatient Clinic at the University of Bergen.

The interview guide was developed in two stages. Initially an outline of the interview guide was developed by the third and fourth authors under the guidance of P. E. Binder who has extensive experience with qualitative approaches. This draft was then further developed in cooperation with the last author, who also has extensive expertise in qualitative research. The first part of the interview guide began with broad and open-ended questions regarding the participants’ experiences with the program as a whole. The next sections of the interview guide included more specific, open-ended questions targeting various aspects of the program. Examples include: “What did you find to be the most useful aspect of Goal Management Training?” and “What was your experience of the group sessions?”.

All interviews were conducted by a member of the research group that the participant had not encountered previously in order to minimize the chance of participants withholding opinions or experiences due to a sense of loyalty to the interviewer. Two interviews were conducted by the fourth author (a clinical psychologist) and eight interviews were conducted by the first author (a psychology graduate student). Both interviewers had training in conducting research interviews and were given guidance from the last author, who is highly experienced in conducting qualitative interviews. The interviews had no set time limit, but the aim was to use 30–60 min per interview. The median interview length was 47 min ranging from 25 min to 82 min. The interviews were transcribed verbatim by the first author, and when speech was unclear, sections or words were marked and looked over by the third author. In order to ensure anonymity, all participants were given an individual ID code, and no names were mentioned in the transcriptions.

### Data analysis

When the first author was conducting the interviews, she became aware of how important the group itself seemed to be to the participants through their frequent mentions of the other group members. This sparked her interest to investigate this further and gave the idea for the analytic focus in the current study. The analysis was therefore data driven, with the analytic focus of the current study being decided after the interview guide was developed. The study thus takes advantage of the opportunities provided by qualitative research to be led by the data to explore aspects that expand the researchers’ preconceptions. However, this also means that there were few explicit questions in the interview guide tapping participants’ experiences of participating in a group-based intervention.

The data material was analyzed using thematic analysis within a hermeneutic phenomenological framework [31, 34]. Nvivo 12 [35] was used as technical support to code (mark and label relevant fragments of text from the interviews) and abstract and organize initial codes into an overarching thematic structure.

In accordance with the six phases of Braun and Clarke, our first step in the analytic work was to familiarize ourselves with the data material [34]. The first, second and last author had the main responsibility for the data analysis. The second author had neither been involved in developing the interview guide nor conducting the interviews, and she thus came from a more neutral standpoint when entering the analytic work. All authors gave written notes with initial impressions and considerations immediately after reading through each interview. Afterwards, all authors had an analysis meeting where we discussed our initial ideas, thoughts and interpretations. The next step in the analysis was to generate codes in relation to the analytic focus (how participants had experienced participating in a group-based intervention), this step was carried out by the first and second author under supervision from the last author. When the coding was completed, we wrote down short summaries containing overall impressions and reflections from each interview. The third step in the analytical work was to start searching for common themes in the interviews. The first and second author read through all of the codes and created a rough thematic map to get a visual representation of our material. We then printed the codes and discussed what we thought they represented and how they connected to one another. As our fourth step, the first, second and last author looked more closely at these initial themes to explore if some of them shared a similar underlying construct or had important commonalities, or if any of the initial themes could better be understood as a sub-theme. In the continuation of reviewing our themes, the first, second, third and last author had a meeting where we discussed the thematic structure. We found that some themes were overlapping and that we could abstract them further. We then defined and named the final themes as a fifth step [34]. The fourth author was not involved in generating the codes and thematic structure, but she was involved in the final stage of finalizing the thematic structure, thus serving the role of an external audit.

### Ethics and reflexivity

The effect study that the data originates from is approved by the Regional Committees for Medical and Health Research Ethics Region West (REK-Vest, 2015/2325). All participants signed an informed consent form. The interviews were audio recorded and the audio files were stored in accordance with ethical guidelines. All identifiable information was removed or anonymized before conducting the data analysis in order to preserve the participants’ anonymity. In addition, all interviews were conducted by a clinical psychologist or a psychology graduate student. The interviewers had been given specific guidelines to follow if serious illness emerged or was uncovered during the interview.

The researchers’ reflexivity is an important principle throughout the research process and has been an important focus in our investigation [31, 34]. The authors made written notes on own preconceptions and expectations going into the project. The authors also held meetings regularly as a team throughout the process, which clarified our own preconceptions. For example, the third and fourth authors are heavily involved in the larger project and have done extensive research within the field of ADHD. The last author, being an experienced qualitative researcher, was attuned to the process of qualitative analysis, and has a keen interest in the client perspective on group therapy but has no previous experience with research on ADHD. Throughout the research process we have paused to discuss our understanding of, and reasons for the choices we have made, using our differences in experience and perspectives actively to facilitate awareness of our own preconceptions, thus reflexive processes.

## Results

The participants differed in how much emphasis they put on the other group members when describing their experiences with GMT. While some found the other group members to be an essential part of their treatment experience, others mentioned it more briefly. Notably, all the participants reported the other group members to be a positive aspect of the treatment, although some descriptions were less rich. Our analysis resulted in one theme capturing the participants’ motivations and expectations prior to treatment, “*The participants’ starting point”*, and two themes capturing the experience of participating in a group-based intervention, “*The ambiguity of the group – the various meanings of the group”* consisting of three sub-themes and “*The group promoted positive change – How the group affected the participants’ everyday lives”* consisting of two sub-themes (see Table 1 for an overview of the themes).

**Table 1:** Overview of main themes and sub-themes

**Main themes**	The participants’ starting point	The ambiguity of the group – the various meanings of the group	The group promoted positive change – How the group affected the participants’ everyday lives
**Sub-themes**		The group created a sense of belonging – “I am not alone”. The personal cost of participating in the group - “At times it was a hot mess”. The group supported the learning experience - “We worked with it together”.	Managing ADHD in daily life - “It’s much easier to handle everyday life”. Personal growth - “Gaining new perspectives".

### The participants’ starting point

We found that participants varied in their expectations, motivations and preconceptions before entering the program. Some reported that they did not have any expectations at all going into the program or that they did not really think much about it beforehand. Some reported that they were curious about the other participants and the group dynamics: “I didn’t really know what to expect, I wasn’t quite sure, like, how it would be to participate in a group conversation with five or six others who have ADHD. Won’t that be very messy, won’t that be chaos?” (Interviewee 3).

Most participants expected that the program would focus on ADHD-related issues and that they would receive some tools that could help them in their everyday lives, while a few expected it would be more similar to “standard” psychotherapeutic group-therapy (e.g., talk about feelings or a mindfulness-based approach).

Many participants were motivated by a wish for help with challenges related to ADHD. Some reported they were motivated by a wish to meet others with ADHD, as it provided an opportunity to hear the experiences of others with the diagnosis: *“*I thought I would spend my time listening and finding out how others with ADHD are doing” (Interviewee 7). One participant reported that the motivation to participate was to contribute to research and help others with ADHD in the future (Interviewee 5).

### The ambiguity of the group – the various meanings of the group

Being in a treatment context with others had both positive and negative aspects. In addition, we found that the same phenomenon could be experienced quite differently by the individual participants. For example, one participant (Interviewee 1) stated that it was annoying that people spoke freely as it made it very hard for this participant to maintain attention in the group, while another participant (Interviewee 3) described it as positive that the participants in the group spoke together so openly. This made us realize how diverse the experiences of the group were. After further examination, this theme appeared to have three distinct sub-themes. These were: *The group created a sense of belonging - “I am not alone”*; *The personal cost of participating in the group - “At times it was a hot mess”*; and *The group supported the learning experience - “We worked with it together”*.

#### The group created a sense of belonging – “I am not alone”

Throughout the data material, there were many statements about the impact of being a part of the group that seemed to involve a feeling of unity - not feeling alone: “Then I thought, well, okay, I am not the only one affected by this, or that I can recognize myself in all these different challenges we face” (Interviewee 8). Through meeting others with the same types of challenges and difficulties, the participants expressed that they felt recognition. This led to feelings of community and affiliation. Being in the group seemed to promote belonging and safety as well as evoking empathy and compassion.

The diminished feeling of being alone did not appear to be related to the actual knowledge that there are others out there with the same diagnosis. Instead, it seemed to be the power of the *actual* meeting, where the participants could see and hear for themselves that there were others in similar situations, that generated this feeling of not being alone: “You get a sense of community, that somehow, you’re not alone [...] Like, you know you’re not alone, but to *actually* meet someone” (Interviewee 2). In addition, there seemed to be a sense of normalization in meeting others with similar experiences: “It’s interesting to hear the experiences of others, both the recognition and also this kind of relief in seeing that others deal with the same things” (Interviewee 5).

Through meeting others in similar situations, one’s own experiences and prejudices were seen from a new perspective, and this seemed to help the participants feel more accepting towards themselves:

Because, when you are diagnosed at my age, you think ‘am I the only one who’s this dumb at this age, that I’m not able to fully take responsibility for my actions?’ That it is excused by something [...] And then one experiences this, that I am not alone here (Interviewee 3).

#### The personal cost of participating in the group - “At times it was a hot mess”

This sub-theme arose from expressions from participants experiencing that the group became somewhat of an obstacle in the training program. Most of these descriptions centered around the other group members distracting them in their learning process, for instance by talking too much, wandering off the main topic, interrupting the other group members or by triggering anxiety. Participants said these kinds of distractions could make them angry or annoyed, or that they lost their concentration when trying to focus on the task at hand:

I sometimes felt, or I many times felt, that it went totally off track. We just talked

about things that weren’t relevant to what we were doing there and then [...] So I felt

that this, this is too much (Interviewee 2).

A few participants also reported that they felt different from people in the group and that this could be burdensome. For instance, one participant mentioned how they sometimes felt like an outsider by not relating to the other participants’ difficulties: “At times I felt a bit different, maybe, compared to many others in the group [...] maybe there were differences in what we were struggling with” (Interviewee 2). On the contrary, other participants would describe the heterogeneity within the group as something positive and something that contributed to a greater outcome of the training program: “It’s good that you have people who are different, because if you were to have a group where everyone’s completely the same, the outcome would have been less, I think” (Interviewee 1).

Even though many described situations in which the group cost them something, there was still a sense of ambivalence in their descriptions, and none of the participants had a solely negative attitude towards the group. One participant described how the in-class exercises had to be excessively explained, which hindered the learning outcome, but at the same time the participant displayed understanding for it having to be like that:

You get an easy task, and then it has to be explained down to the tiniest detail what we’re going to do, right? But it’s just that, everyone has different skills, and there might be things I don’t understand that easily (Interviewee 1).

#### The group supported the learning experience - “We worked with it together”

The third sub-theme demonstrates how the participants experienced the group to be a support in their learning experience during the treatment. There were several reports of the program being more interesting when conducted in a group, enhancing the participants’ focus and attention, and that it was easier to understand the tasks based on other group members examples and input. Through the discussions they would learn from each other, and they had to be considerate of one another. It all seemed to affect their learning outcome. Just being together with others made one feel encouraged to work in the program: “The cooperation that we had in the group; now we are going to do something, now we are together” (Interviewee 9).

Some participants reported that the program was more comprehensible because it was conducted in a group: “I think it would have been very boring, monotonous and much more difficult to do something tangible and useful if I just sat in a room by myself or just read it” (Interviewee 6). Many participants also reported that it was not necessarily the assignments themselves that were crucial, but discussing them together in a group: “Of course we understand the point with the different assignments quite quickly, but discussing how the assignments were and what they did to us is so unbelievably interesting” (Interviewee 3). It became apparent that the participants helped each other’s learning experience in several ways, and that they appreciated the possibility to share experiences with the other group members: “To be able to reflect upon and discuss our experiences along the way was very useful, to share them” (Interviewee 4). Learning from each other and together seemed to be a central part of the participants’ experiences and also seemed to be an important aspect to most of the participants.

### The group promoted positive change - how the group affected the participants’ everyday lives

Many participants described how the group had impacted their lives outside the program and further improved their day-to-day life. The discussions and exchange of experiences that took place within the group sessions were often described as a significant influence for positive changes occurring in the participants’ lives. Many of these changes were naturally centered around ADHD, as GMT encourages participants to share coping techniques and positive experiences related to this. More surprisingly, participants also described how the group had contributed to a positive change on a more personal level, such as becoming more confident or finding a new acceptance for oneself or towards others. This theme has two separate sub-themes: *Managing ADHD in daily life - “It’s much easier to handle everyday life”*, and *Personal growth - “Gaining new perspectives”*.

#### Managing ADHD in daily life - “It’s much easier to handle everyday life”

Many participants described how the other group members’ experiences were a valuable resource when coping with their own symptoms of ADHD: “Sharing stories and experiences about ADHD, and in a way, coping with ADHD in daily life has perhaps become a bit easier after having taken part in this” (Interviewee 8). Members of the group would share experiences and coping techniques that the participants, in turn, could integrate into their own lives. The participants also mentioned how it was easier to cope with and accept the mistakes they made due to their ADHD symptoms, when learning that others too had similar experiences.

Many participants emphasized the heterogeneity of ADHD and within the group. Regardless, some of the participants expressed that even though the group members had different challenges, the input of the other members was still useful: “Everyone has their difficulties, and they can be very different even though you have ADHD, but then there’s that, that the same techniques others are using I can use myself” (Interviewee 3).

Some participants also perceived the members of the group to have more credibility than the healthcare professionals, as they had personal experience with these issues themselves and thus their input weighed more heavily. The group could offer some advice that the healthcare professionals simply were not able to: “It’s probably the users who can tell you the most about an experience, not the ones prescribing the medication” (Interviewee 2).

The sub-theme illustrates how the group was helpful in adjusting to and coping with ADHD in daily life. The participants valued the input and advice from others who also had ADHD and reported this to be of significance to their increased coping.

#### Personal growth - “gaining new perspectives”

The findings indicate that the group also contributed to a personal growth that was not specifically related to coping with ADHD, but rather to a positive change in how they perceived themselves or others. By meeting others and sharing experiences the participants were able to see things from another angle or gain new insight.

Some participants reported that meeting others with ADHD had a positive impact on their thoughts and feelings about themselves: “I notice that it helps a lot when it comes to what I think about myself [...] I don’t feel as bad about myself, because I know that there are many others who have it” (Interviewee 10). Some participants also experienced that meeting others contributed to a better understanding of themselves: “I think that meeting others in a similar situation with a similar problem has been a very interesting way of shedding light upon my own, where I stand kind of [...] I see myself better, in a way” (Interviewee 6).

Some participants also mentioned how the group played a role in them having a greater understanding for other people and others with ADHD:

I have a son who has ADHD as well, and our ADHD is very different from one another, so I feel that I understand him better now. Because well yeah, I have always understood that he has ADHD and that he has his challenges related to that, but I never understood …. that *that’s* been a part of his challenges, like the different aspects of it. But after meeting all these different people that I have met here, I now see that ‘oh, that’s a part of his ADHD’ (Interviewee 1).

This final sub-theme indicates that the other group members were not only significant for coping with ADHD, but the group was also described to contribute to personal growth, increasing positive thoughts and feelings about themselves and expanding their understanding of others.

## Discussion

The aim of the current study was to explore how adults with ADHD experienced participating in a group-based intervention (GMT) for ADHD. Although participants entered treatment with rather varying degrees of specific expectations, as illustrated in theme 1, most hoped to get specific tools that could help them better manage their everyday life living with ADHD. Their hopes for treatment outcomes can therefore be seen as quite instrumental. Although knowing they would participate in a group-based intervention, the group as such was not frequently in the participants’ focus when entering treatment. However, after having completed the GMT training, receiving treatment in a group was meaningful in its own right for these participants. Moreover, participation in the group had given many of the participants something they had not explicitly hoped for before entering treatment – they had experienced personal growth, in addition to an improved management of ADHD in their everyday life. Their perspectives had expanded, whether it was seeing oneself or others in different light, feeling normalized, or findings new ways to cope with ADHD.

How, then, can we understand the participants’ experiences? And what role did the group play in facilitating them? One of the most prominent findings was that the participants experienced recognition and a sense of belonging in the group. This experience did not seem to be related to the knowledge or discovery that there were others who struggled with similar difficulties, but rather related to the immediate feelings and experiences that emerged when meeting others in the group. Individuals with ADHD are often met with stigma and prejudice, which may induce feelings of shame and lead to an experience of being different to others [5, 36]. Hearing about other people’s experiences often allows people to feel more part of humankind and thus less alone [37]. The experiences and stories of the other group members allowed for the individual participants to expand their perspectives, as well as build acceptance toward themselves and their life situations. Unlike many situations, where the participants’ struggles with tasks, like organization and regulation, may lead to negative feedback, the group setting provided a forum where one’s struggles were understood and shared by the other group members, thus an opportunity to reflect upon one’s struggles and possible resources to increase one’s coping of them.

Many participants incorporated the advice and coping strategies of the other group members into their everyday lives. This finding is in accordance with previous research on other patient groups, where it has been found that participants in group-based interventions incorporate the successful behaviors and attitudes of other group members into their own lives [38]. The participants also valued that the other group members had personal experience with similar challenges as themselves, unlike most healthcare professionals. This kind of user experience is often viewed as therapeutic and helpful [39]. This finding is in accordance with knowledge we have about the impact of *who* says or does something on *how* it is perceived [40]. Interaction with similar others also enhances modeling behavior and group cohesiveness, removes feelings of isolation, and motivates change [38, 41]. These findings indicate that incorporation of peers in treatment for adults with ADHD might be therapeutic and helpful.

It is interesting, then, to look at the interplay between the opportunity to meet others in similar situations and the structure provided by the GMT program in facilitating the above-mentioned experiences. While meeting others and exchanging experiences was underlined by the participants, and has also previously been reported in research on group-based interventions for ADHD [20], the group was also experienced to hinder learning at times, with other group members being experienced as both annoying and disturbing. This points to the importance of combining structure, including a group therapist facilitating interaction between group members, maintaining focus, and introducing structured exercises, with the opportunity for group members to exchange experiences and utilize the unique opportunity provided in the group to stop and reflect on their challenges and ways to handle them.

Moreover, in a group setting, group members have to both give and receive in order to make the group work. Yet, when describing their experiences, the participants mainly focused on what they had received, not their own contributions to the group. Allowing oneself to receive input from others is essential in order to expand one’s perspective. When the participants heard the experiences and perspectives of the other group members, they were able to step out of their own shoes, thus allowing for their own perspective to be broadened. The findings thereby shed light on how the group therapeutic factors of universality, group-cohesion and hope [37] might be particularly important for adults with ADHD, because it represents an antidote to stigma and prejudice and gives an unique opportunity to meet others within a supportive structure, thus experiencing personal growth in addition to an improved management of everyday life.

In contrast to the above-mentioned findings, some participants did not have the same experience of recognition and relatedness. Research on group therapy shows that more homogenous groups benefit more from group therapy than those of mixed symptoms [42, 43]. Because of the heterogeneity in ADHD and its high comorbidity rates, the participants were likely to have a variety of symptoms and challenges, which might have contributed to difficulties in relating to the other group members [44, 45]. Not experiencing recognition in the group may have led some participants to maintain feelings of otherness and aloneness [37, 46]. On the other hand, some participants described the group’s heterogeneity to be a positive aspect, as it enhanced their understanding for others and in turn increased their outcome of the treatment. Hence, although it might be challenging to be in a group with people who have different challenges, symptoms and needs, facing such challenges might also expand one’s perspective and perhaps result in more tolerance towards others. This finding does, however, point to the vigilance group therapists must show when making decisions on group compositions, and the challenge the heterogeneity of ADHD symptoms represents when attempting to utilize the potential of group-based interventions in treating adults with ADHD.

### Implications

The findings demonstrate how incorporation of peers in treatment for adults with ADHD can lead to favorable, but also unfavorable, outcomes, underscoring a need to tailor the treatment to meet the client’s needs. These findings may suggest that monitoring of the clients’ learning outcome might be an important step towards facilitating learning in a group. A more coherent picture of how the group mediates or moderates learning could help direct solutions to optimize learning in a group. The participants in the study also described how the act of sharing personal experiences and coping strategies in the group was highly appreciated. This finding suggests that it might be beneficial to facilitate an open and helping environment in the group in order to promote supportiveness and self-disclosure among the participants.

As evident from the experiences of the participants, finding a fitting balance between a structure that allows for both individual learning of the content of the intervention and the open-ended discussions which the participants appreciated may be a central aim for future designs. Participants both described challenges related to discussions going “off track”, while maintaining that some of the most useful insights of participation were a result of seeing both similarities and differences between their own perspectives and the perspectives of the other group members.

Relating to the above-mentioned challenge, it was also found that some experienced recognition in the group, while others did not. Implications of this may be to design group-based interventions in such a fashion that it targets themes and issues that are broad enough to relate to as many group members as possible. This may ensure group cohesion and may also prevent participants from perceiving the intervention as irrelevant or inadequate.

### Limitations

The current study has several limitations. First, only half of the participants that completed the GMT program wanted to participate in the qualitative interview. Therefore, there is a chance that those who agreed to be interviewed were more satisfied with the program or in other ways differed from those who did not partake. The sample also mostly consisted of males, which might have implications for transferability of the results. Another limitation is that the interview guide was not developed to specifically fit the current analytic focus. Although there were several open-ended questions, there were only a few questions asking specifically about the experiences of the group participation. This might have led to certain group experiences being omitted as a result of the interviewer not asking more detailed questions. At the same time, the aspect of the other group members came up repeatedly, even when the focus of the interview was not specifically on this, which might indicate that the group itself was a central part of the participants’ experiences. Moreover, our own pre-knowledge and preconceptions also influence the findings. All of the researchers are trained as clinical psychologists which affects the way we understand and interpret the data material. Specifically, as clinical psychologists we are likely to have a more positive attitude towards psychotherapy, while for example being more hesitant towards pharmacological treatment. In an attempt to limit the influence of such preconceptions, we have worked actively with reflexive processes throughout the research process, as detailed in the reflexivity statement in the methods section.

### Future directions

The findings from the current study indicate that future research should aim at exploring more thoroughly the characteristics of those benefitting and not benefitting from group-based interventions for ADHD, concurrent to pharmacological treatment or not. We also suggest that future research should utilize process-oriented research in order to investigate how clinicians can recognize those who are not benefitting from group-based approaches, and consequently how these individuals may be helped. We outline that it may be important for future research to have larger sample sizes, and utilize complementing methodologies, especially when investigating what factors are decisive for improvement and deterioration in group-based interventions for adults with ADHD.

## Conclusion

The aim of the current study was to explore how adults with ADHD experienced participating in a group-based intervention (GMT) for ADHD. Overall, the findings underscore how valuable the group itself may be to the individual participant. The participants reported that meeting others with similar challenges created an experience of recognition, as well as a sense of belonging and community. The group was also reported to contribute to better coping with ADHD, as well as personal growth, where the exchange of coping strategies and experiences were seen as important and meaningful. It was, however, also found that the group was perceived as a burden at times, for instance by increasing feelings of being different or making one lose focus on the training. In conclusion, being in a group could both be challenging and rewarding. However, despite the challenging aspects of being in a group, most participants found the group participation to be meaningful and helpful, with the group being an advantage in their process of change.

## Supplementary Information

**Additional file 1.** Interview protocol (English).

## Data Availability

The datasets/interview transcripts are not available to the public due to ethical considerations and in order to preserve the participants’ privacy. The datasets used in the study are available from the corresponding author on reasonable request.

## Abbreviations

ADHD: Attention Deficit Hyperactivity Disorder
GMT: Goal Management Training
