# Supplementary material for: Goal management training for adults with ADHD – clients’ experiences with a group-based intervention
Source: BMC Psychiatry. 2021 Feb 19;21:113. doi: 10.1186/s12888-021-03114-4 (PMC7893765; doi:10.1186/s12888-021-03114-4)
Supplement: Supplementary file 1 — Additional file 1. Interview protocol (English). [file 12888_2021_3114_MOESM1_ESM.docx]

**Interview protocol (English)**

I would like to talk to you about how you experienced the group sessions you have participated in. I also have some questions regarding your experience with Goal Management Training, what positive and negative experiences you have had, and what you have experienced as helpful, or not helpful.

I would like to begin with a couple of general questions, and then I will ask some specific questions as we go along.

1. First, could you tell me a bit about how you experienced taking part in Goal Management Training?
2. What did you hope that Goal Management Training could help you with?
3. Now I would like to ask you some more specific questions, and the first thing I would like to ask you is what did you experience as the most useful for you?

*If affirmative answer:*

- 1. Can you recall a situation from the sessions that was particular positive for you? (what was the situation, what happened, what did you feel/think, what significance did it have for you?)
  2. Can you recall a situation from everyday life where you experienced that you benefitted from what you learned during your participation? (what was the situation, what happened, what did you feel/think, what significance did it have for you?)

*If not affirmative answer:*

- 1. Do you have some thoughts on why you did not experience this treatment offer as useful?

1. Was there anything about the treatment offer that you experienced as difficult or challenging? Is there anything that you would describe as a disadvantage of having participated?
   1. Can you recall a situation from the sessions that was particular difficult or challenging? (what was the situation, what happened, what did you feel/think, what significance did it have for you?)
2. How did you experience participating in the sessions?

*(If the participant does not compare tutoring, assignments and the discussions parts, ask if she/he had a different outcome of these).*

1. Can you tell me a bit about how you experienced the exercises and strategies that we have focused on in the sessions?
   1. *How does the participant experience that the exercises/strategies have affected oversights and inattentive errors in everyday life?*
   2. *Has the use of these affected other aspects of daily living (distress for such errors, trust in own abilities?)*
   3. *Does the participant distinguish between the exercises (attentive presence) and the STOP-strategies? Does the participant use these in different situations/for different reasons?*
2. Can you tell me a bit about how it was to use the workbook between the sessions?
   1. *Did the participant find this to be a useful tool in everyday life?*

*If yes:*

- - 1. *How did she/he use the workbook?*

*If no:*

- - 1. *What was the reason for this?*

1. If we were to extend the treatment offer with 4-5 sessions, what would you like to fill those with?
   1. *Examples of themes, exercises or discussion the participant wished there were more of?*
   2. *Examples of themes, exercises or discussion the participant missed?*
2. Do you experience that anything has changed after participating in this project?

*If yes:*

- 1. *What does these changes consist of?*
  2. *In what ways is this different now from before?*
  3. *Does the participant connect these changes to the focus in Goal Management Training (exercises, strategies, awareness of own challenges), or other factors (sharing experiences, social contact, connectedness?)*
  4. *Could you tell me about a situation that you experience as a good example of this change? What do you think you do differently now?*
  5. *Are these changes present in various situations? Work, socially, leisure time, or in relation to how the individual perceive herself/himself?*

*If no:*

- 1. *Do you have any thoughts on why you have not experienced any changes after the training program?*

*If not covered in the sections above:*

- 1. *Following the training program, do you experience any changes in how you perceive tasks or projects that you are to, or want to, complete? (Does the participant connect these changes to the focus in Goal Management Training (exercises, strategies, awareness of own challenges), or other factors (sharing experiences, social contact, connectedness?)).*

1. What are your thoughts on offering Goal Management Training to others who are experiencing similar challenges as yourself? For instance, would you recommend Goal Management Training to a friend or a family member?
2. Now that you have completed the treatment, I am also wondering if you believe that use of technology, for instance an app on mobile phones, would have affected your outcome of Goal Management Training?

*If affirmative answer:*

- 1. Do you have any thoughts on what functionalities would be particular useful for you in such an app?

*If not affirmative answer:*

- 1. What is the reason that you think that such a tool would not have affected your outcome?

1. Lastly, I am wondering if there are any other things that are important to you that I forgot to ask about, or that you would like to say something about before we finish?
